
JOURNAL INFORMATION
==============================
NLM Title Abbreviation: Biospektrum (Heidelb)
Journal ID: Biospektrum (Heidelb)
NLM Journal ID: 9615685

Biospektrum

ISSN: 0947-0867
EISSN: 1868-6249

ARTICLE INFORMATION
==============================
PMCID: PMC8960675
PMID: 35369109
DOI: 10.1007/s12268-022-1742-5
Article ID: 1742
Article version: 1
Subjects: Wissenschaft

Mitochondrien als universelle Sensoren der akuten Hypoxie?

Nolte Anika
Pak Oleg
Sommer Natascha Natascha.Sommer@innere.med.uni-giessen.de

grid.8664.c 0000 0001 2165 8627 Exzellenzcluster „Cardiopulmonary Institute“ Lungenzentrum der Universität Gießen und Marburg (UGMLC), Mitglied des Deutschen Zentrums für Lungenforschung (DZL), Justus-Liebig-Universität, Aulweg 130, D-35392 Gießen, Deutschland
Electronic publication date: 2022 Mar 28
Print publication date: 2022
Volume: 28
Issue: 2
First page: 132
Last page: 134
Copyright: © Die Autorinnen und Autoren 2022
License: Open Access: Dieser Artikel wird unter der Creative Commons Namensnennung 4.0 International Lizenz veröffentlicht, welche die Nutzung, Vervielfältigung, Bearbeitung, Verbreitung und Wiedergabe in jeglichem Medium und Format erlaubt, sofern Sie den/die ursprünglichen Autor(en) und die Quelle ordnungsgemäß nennen, einen Link zur Creative Commons Lizenz beifügen und angeben, ob Änderungen vorgenommen wurden. Die in diesem Artikel enthaltenen Bilder und sonstiges Drittmaterial unterliegen ebenfalls der genannten Creative Commons Lizenz, sofern sich aus der Abbildungslegende nichts anderes ergibt. Sofern das betreffende Material nicht unter der genannten Creative Commons Lizenz steht und die betreffende Handlung nicht nach gesetzlichen Vorschriften erlaubt ist, ist für die oben aufgeführten Weiterverwendungen des Materials die Einwilligung des jeweiligen Rechteinhabers einzuholen. Weitere Details zur Lizenz entnehmen Sie bitte der Lizenzinformation auf http://creativecommons.org/licenses/by/4.0/deed.de.
License URL: https://creativecommons.org/licenses/by/4.0/

issue-copyright-statement: © The Author(s), under exclusive licence to Springer-Verlag GmbH Deutschland, ein Teil von Springer Nature 2022

==============================
Adaptation to acute hypoxia through cardiorespiratory responses is mediated by specialized cells in the carotid body and pulmonary vasculature to optimize systemic arterial oxygenation. Acute oxygen sensing thus is a fundamental pre-requisite for aerobic life. Recent studies unravelled basic oxygen sensing mechanisms involving the mitochondrial cytochrome c oxidase subunit 4 isoform 2 that regulates the release of mitochondrial reactive oxygen species and subsequent acute hypoxic responses.

■ Sauerstoff ist zum Überleben der meisten Lebewesen und des Menschen unverzichtbar. Eine auch nur leicht verminderte Konzentration des Sauerstoffgehalts (Hypoxie) in der systemischen Zirkulation oder der Lunge wird daher in wenigen Sekunden von spezialisierten Zellen wahrgenommen und löst adaptive Antworten aus [1, 2]. Eine systemische arterielle Hypoxie wird in Chemorezeptoren des *Glomus caroticum* (Carotis-Körper) in der Carotis-Verzweigung der Halsschlagader detektiert und führt zur rapiden Adaptation über kardiorespiratorische Reflexe (Hyperventilation und Aktivierung des Sympathikus) [1]. Dabei werden unter Hypoxie, auch im milden Bereich, plasmalemmale, spannungsabhängige Kaliumkanäle gehemmt, wodurch die Zellen der neurosensorischen Carotis-Körper depolarisiert werden und einen Einstrom von Calcium auslösen. Die darauffolgende Freisetzung von Transmittern aktiviert die afferenten Nervenzellen und stimuliert das respiratorische und autonome Zentrum des Hirnstamms [1]. Der primäre Sensormechanismus der Hypoxie als Trigger für diese Signalkaskade in diesen spezialisierten Zellen des *Glomus caroticum* ist jedoch nicht aufgeklärt. Weiterhin verfügt die Durchblutung der Lunge und Plazenta über spezielle Adaptationsmechanismen an akute Hypoxie, um die $O_2$-Versorgung des jeweiligen Organismus sicherzustellen, deren primärer Sensormechanismus ebenfalls Gegenstand aktueller Forschung ist [2].

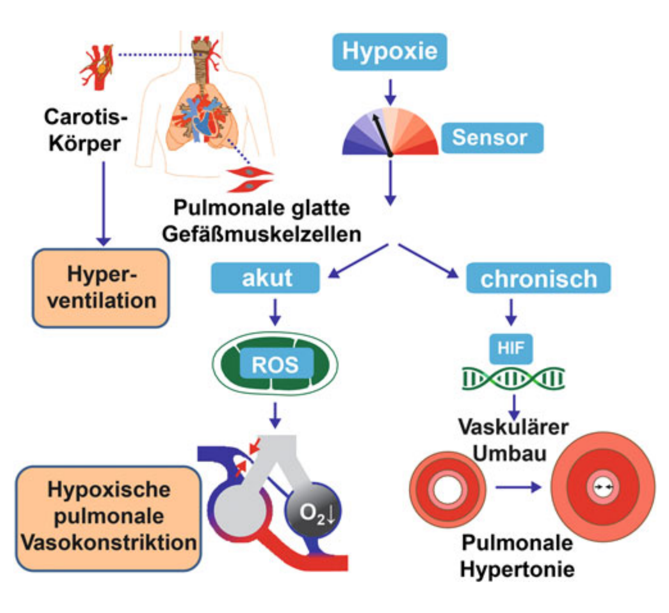

◀ **Abb. 1:** Zelluläre Hypoxiesensoren. Akute Hypoxie wird im Carotis-Körper und in glatten Gefäßmuskelzellen der Lunge detektiert und führt zu Anpassungsmechanismen, wie Hyperventilation und hypoxischer pulmonaler Vasokonstriktion. Chronische Hypoxie unterliegt teilweise anderen Sensormechanismen und führt zum pulmonalvaskulären Gefäßumbau. ROS: reaktive Sauerstoffspezies; HIF: Hypoxie-induzierter Faktor.

### Die hypoxische pulmonale Vasokonstriktion

In der Lunge wird ein Sauerstoffmangel in den Lungenbläschen (Alveolen) durch die glatten Muskelzellen der Lungengefäße detektiert. Akute alveoläre Hypoxie löst dadurch eine Kontraktion der kleinen präkapillären pulmonalen Arterien aus, sodass die Lungendurchblutung in Bereichen mit vermindertem alveolären Sauerstoffgehalt eingeschränkt wird, wodurch der Gasaustausch in der Lunge optimiert wird [2]. Dieser Mechanismus wurde erstmalig 1946 von von Euler und Liljestrand beschrieben und auch als hypoxische pulmonale Vasokonstriktion (HPV) bezeichnet [3]. Der molekulare Sauerstoffsensor befindet sich in den glatten Gefäßmuskelzellen und ist ähnlich wie der des Carotis-Körperchens nicht vollständig aufgeklärt (**Abb. 1**).

Unter bestimmten Bedingungen kann die HPV eingeschränkt sein und zu einer systemischen Hypoxie führen. Zu diesen zählt z. B. eine Entzündung der Lunge bis zum akuten Lungenversagen (*acute respiratory distress syndrome*, ARDS), das hepatopulmonale Syndrom oder eine Anästhesie. Eine ungleichmäßige HPV trägt auch zur Entwicklung eines Höhenlungenödems bei [2].

Eine andauernde HPV unter chronischer Hypoxie kann dagegen zur Entstehung einer pulmonalen Hypertonie beitragen, die durch Gefäßumbauprozesse verursacht wird und zu einer Rechtsherzbelastung führt. Die Sensor- und Regulationsmechanismen von pathologischen Prozessen während langanhaltender und chronischer Hypoxie überschneiden sich allerdings nur teilweise mit denen der akuten physiologischen Adaptationsmechanismen [2]. Dementsprechend kann die HPV in eine akute Phase (Sekunden bis Minuten anhaltende Vasokonstriktion), die die Durchblutung von einem Atemzug zum nächsten an die alveoläre Sauerstoffkonzentration anpasst, und in eine länger andauernde Phase (Minuten bis Stunden anhaltende Vasokonstriktion) unterteilt werden [2]. Um therapeutische Strategien zur Verbesserung einer eingeschränkten physiologischen HPV oder übersteigerten pathologischen HPV zu entwickeln, ist das Verständnis der zugrunde liegenden molekularen Mechanismen, insbesondere auch des primären Sauerstoffsensors, von enormer Bedeutung.

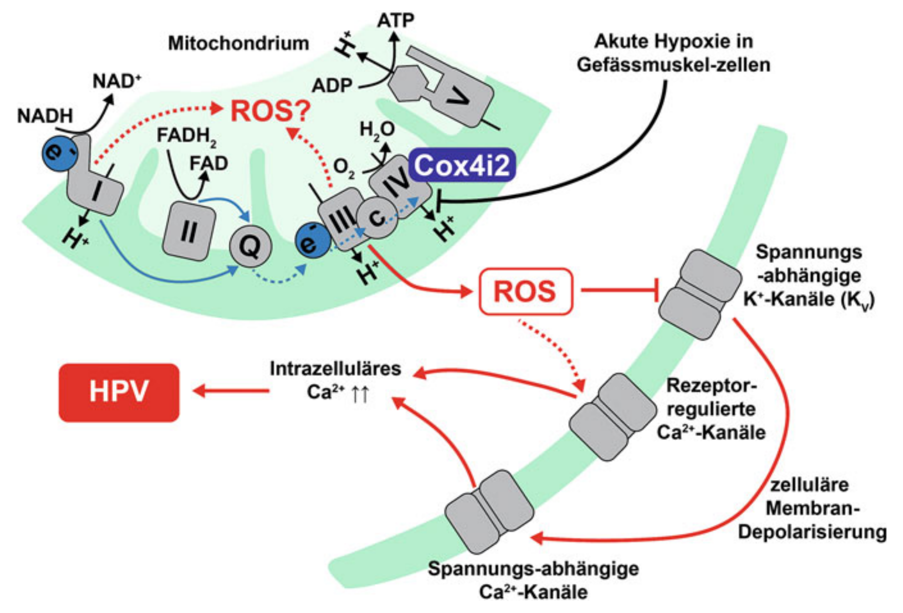

▲ **Abb. 2:** Sensor- und Signaltransduktion der akuten hypoxische pulmonalen Vasokonstriktion (HPV). Akute Hypoxie induziert in pulmonalen glatten Gefäßmuskelzellen eine Cox4i2-abhängige Freisetzung von reaktiven Sauerstoffspezies (ROS) aus der Atmungskette, die über Interaktion mit Kalium($K^+$)- und Calcium($Ca^{2+}$)-Kanälen zu einem Anstieg des intrazellulären $Ca^{2+}$ und auf Organebene zu der HPV führt.

## Mitochondrien als Sensoren der akuten Hypoxie

Mitochondrien dienen zur Generierung von Adenosintriphosphat (ATP) als zellulärem Energieträger. Dazu wird die Energie, die bei der Übertragung eines Elektrons entlang der Elektronentransportkette (ETC) frei wird, genutzt, um einen Protonengradient an der mitochondrialen Membran aufzubauen, der die ATPase antreibt. Das Elektron wird dabei von Komplex IV (Cytochrom-c-Oxidase) der Elektronentransportkette auf Sauerstoff als finalem Elektronenakzeptor übertragen. Reaktive Sauerstoffspezies (ROS) können als Nebenprodukte der Elektronentransportkette vor allem an Komplex I und III entstehen. Neben der Funktion als Signalmoleküle in biochemischen Prozessen, können hohe Konzentrationen von ROS Zellschädigungen verursachen. Die Bedeutung der Mitochondrien bei der Detektion der akuten Hypoxie und der mitochondrialen ROS als Signalmoleküle ist seit langem bekannt, der primäre Sauerstoffsensormechanismus blieb allerdings lange Zeit unklar. Aus bisherigen Untersuchungen an Mitochondrien aus anderen Organen wurde angenommen, dass die Cytochrom-c-Oxidase nicht als primärer Sauerstoffsensor der akuten Hypoxie in Zellen des *Glomus caroticum* oder der Lungengefäße dienen könnte, da aufgrund ihrer hohen Sauerstoffaffinität die Sensitivität gegenüber milder Hypoxie zu gering wäre [2].

## Die Rolle von Cox4i2 beim Sauerstoffsensormechanismus

Mit der Identifikation der Isoform 2 der Untereinheit 4 der Cytochrom-c-Oxidase (Cox4i2), die relativ stark in den Lungengefäßen exprimiert wird, wurde allerdings die Hypothese aufgestellt, dass eine spezielle Zusammensetzung der Cytochrom-c-Oxidase in glatten pulmonalen Gefäßmuskelzellen deren Sensitivität gegenüber Hypoxie erhöhen könnte [4]. Darauffolgende Untersuchungen ergaben, dass in Mäusen mit einem Knockout für diese Untereinheit die akute HPV und Hypoxie-induzierte Hyperventilation fehlte, während die pulmonale Vasokonstriktion und Hyperventilation auf andere Stimuli vollständig erhalten blieb [1, 5]. Als zugrunde liegender Mechanismus zeigte sich eine Inhibierung der Hypoxie-induzierten Freisetzung von mitochondrialem Superoxid in glatten Gefäßmuskelzellen der Cox4i2-Knockout-Mäuse. Es wurde in diesen Untersuchungen gezeigt, dass unter physiologi-

Hier steht
eine Anzeige.
Springer

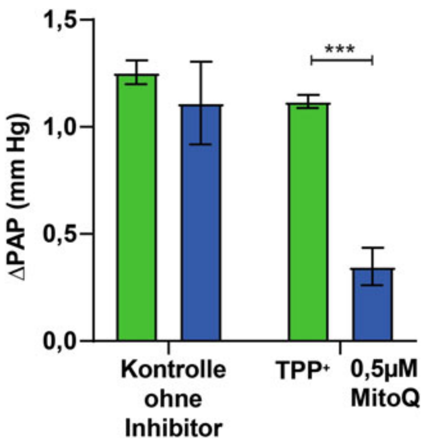

▲ **Abb. 3:** Inhibierung der hypoxischen pulmonalen Vasokonstriktion (HPV) durch das mitochondriale Antioxidant MitoQ. MitoQ inhibiert den Anstieg des pulmonalarteriellen Drucks (ΔPAP), der durch eine hypoxische Beatmung in der isolierten Lunge ausgelöst wird. MitoQ besteht aus einer Ubichinon-Einheit, die zur Anreicherung in Mitochondrien an Triphenylphosphonium (TPP+) gebunden ist, das als Kontrollsubstanz verwendet wurde. Adaptiert aus [7].

schen Bedingungen Wasserstoffperoxid, das aus Superoxid entstehen kann, mit Ionenkanälen der Zellmembran interagiert und Kaliumkanäle inhibiert. Die Folge ist eine Hypoxie-induzierte Depolarisierung der Zellmembran, Aktivierung von spannungsabhängigen Calciumkanälen und schließlich ein intrazellulärer Anstieg von Calcium. Dies führt zu einer Kontraktion der präkapillaren pulmonalen Arterien und HPV [5]. Ähnliche intrazelluläre Mechanismen wurden für die Cox4i2-abhängigen Signalwege im *Glomus caroticum* gezeigt [1]. Der genaue Mechanismus der Cox4i2-abhängigen ROS-Freisetzung ist noch unklar, es konnte aber gezeigt werden, dass möglicherweise eine Elektronenakkumulation an Komplex III der Atmungskette die Superoxidfreisetzung fördert (**Abb. 2**, [6]). Wie bereits früher gezeigt, unterscheiden sich allerdings akute und chronische Hypoxie-Sensormechanismen in den Lungengefäßen. Entsprechend hatte der Cox4i2-Knockout kaum Einfluss auf die Entstehung der chronischen Hypoxie-induzierten pulmonalen Hypertonie [5].

Die Aufdeckung dieses Signalwegs kann möglicherweise als therapeutischer Ansatzpunkt dienen, z. B. durch Unterstützung der physiologischen HPV bei Lungenentzündung, aber auch durch Inhibierung einer übersteigerten HPV, die zur pulmonalen Hypertonie beiträgt, z. B. durch Einsatz von ROS-Inhibitoren (**Abb. 3**, [7]). Die Rolle der mitochondrialen ROS-Freisetzung als universeller akuter Sauerstoffsensormechanismus und die Bedeutung von Cox4i2 bei der mitochondrialen Superoxidfreisetzung in anderen Zelltypen muss weiter untersucht werden.

## Fazit

Um therapeutische Strategien für pathologische Zustände, in denen die HPV eingeschränkt oder übersteigert ist, zu entwickeln, ist das Verständnis der Sensor- und Signalmechanismen, die der HPV zugrunde liegen ausschlaggebend. Dies trifft zurzeit mit besonderem Augenmerk auf Patient:innen zu, die während der SARS-CoV-2-Pandemie an einer COVID-19-Pneumonie erkranken, die mit prominenten pulmonalvaskulären Veränderungen und sowohl eingeschränkter HPV, als auch pulmonaler Hypertonie einhergehen, die zu schwerer systemischer Hypoxie und Rechtsherzbelastung führt [8]. ■


Funding note: Open Access funding enabled and organized by Projekt DEAL.

Anika Nolte 2012–2017 Biologiestudium an der Universität Gießen mit den Schwerpunkten Mikrobiologie und Biochemie. Ab 2017 anschließende Promotion am Exzellenzcluster „Cardiopulmonary Institute“ zu dem Thema „Die Rolle von Cox4i2 als akuter Sauerstoffsensor“.

Oleg Pak Kardiologe und Scientist, seit 2007 am Exzellenzcluster „Cardiopulmonary Institute“ tätig. Die wissenschaftliche Arbeit fokussiert sich auf die Rolle der Mitochondrien bei physiologischen und pathologischen Prozessen in pulmonalen Zellen und in der Pathogenese von pulmonaler Hypertonie und COPD.

Natascha Sommer Pneumologin und Clinician Scientist mit Schwerpunkt Lungenhochdruck und COPD. Seit 2010 Leiterin einer Arbeitsgruppe zur mitochondrialen (Patho-)Physiologie in der Lunge am Exzellenzcluster „Cardiopulmonary Institute“. 2018 Forschungspreis der Deutschen Gesellschaft für Pneumologie für Arbeiten zur Rolle von reaktiven Sauerstoffspezies und Mitochondrien bei Lungengefäßerkrankungen.


Literatur

[1] Moreno-Domínguez A Ortega-Sáenz P Gao L Acute O2 sensing through HIF2α-dependent expression of atypical cytochrome oxidase subunits in arterial chemoreceptors Sci Signal 2020 13 eaay9452 10.1126/scisignal.aay9452 31848220
[2] Sommer N Strielkov I Pak O Oxygen sensing and signal transduction in hypoxic pulmonary vasoconstriction Eur Respir J 2016 47 288 303 10.1183/13993003.00945-2015 26493804
[3] Von Euler US Liljestrand G Observations on the pulmonary arterial blood pressure in the cat Acta Physiologica Scandinavica 1946 12 301 320 10.1111/j.1748-1716.1946.tb00389.x
[4] Hüttemann M Lee I Gao X Cytochrome c oxidase subunit 4 isoform 2-knockout mice show reduced enzyme activity, airway hyporeactivity, and lung pathology FASEB J 2012 26 3916 3930 10.1096/fj.11-203273 22730437 PMC3425824
[5] Sommer N Hüttemann M Pak O Mitochondrial complex IV subunit 4 isoform 2 Is essential for acute pulmonary oxygen sensing Circ Res 2017 121 424 438 10.1161/CIRCRESAHA.116.310482 28620066 PMC5544581
[6] Sommer N Alebrahimdehkordi N Pak O Bypassing mitochondrial complex III using alternative oxidase inhibits acute pulmonary oxygen sensing Sci Adv 2020 6 eaba0694 10.1126/sciadv.aba0694 32426457 PMC7159913
[7] Pak O, Scheibe S, Esfandiary A et al. (2018) Impact of the mitochondria-targeted antioxidant MitoQ on hypoxia-induced pulmonary hypertension. Eur Respir J 170102410.1183/13993003.01024-2017 29419444
[8] Gierhardt M Pak O Walmrath D Impairment of hypoxic pulmonary vasoconstriction in acute respiratory distress syndrome Eur Respir Rev 2021 30 210059 10.1183/16000617.0059-2021 34526314 PMC9489056
